# Red Wine Oxidation Characterization by Accelerated Ageing Tests and Cyclic Voltammetry

**DOI:** 10.3390/antiox10121943

**Published:** 2021-12-03

**Authors:** Stacy Deshaies, Luca Garcia, Frédéric Veran, Laetitia Mouls, Cédric Saucier, François Garcia

**Affiliations:** SPO, Université de Montpellier, INRAE, Institut Agro, 34000 Montpellier, France; stacy.deshaies@umontpellier.fr (S.D.); luca.garcia@umontpellier.fr (L.G.); Frederic.veran@inrae.fr (F.V.); laetitia.mouls@supagro.fr (L.M.); cedric.saucier@umontpellier.fr (C.S.)

**Keywords:** cyclic voltammetry, phenolic compounds, red wine, oxygen consumption rate, oxidation

## Abstract

In order to obtain information on the oxidative behavior of red wines, oxygen consumption rates and electrochemical changes (cyclic voltammetry) were measured for nine red wines subject to three different accelerated ageing tests: chemical (with hydrogen peroxide), enzymatic (with laccase from *Trametes versicolor*), and temperature (at 60 °C). Oxidative behavior depended both on the wine sample and accelerated ageing test type. A good correlation was observed between electrochemical parameters of charges for reference/non-oxidized wines, in accordance with their antioxidant capacity, and the variation of charges after enzymatic and temperature tests, meaning that cyclic voltammetry could be used in order to predict these two oxidation tests and reflect the wine sensitivity towards respective oxidation targets. However, it was not possible to predict wine chemical oxidation test based on hydrogen peroxide from the electrochemical measurements.

## 1. Introduction

During the winemaking process and storage, red wines can undergo many undesirable changes, particularly oxidative degradation due to numerous atmospheric oxygen intakes. This spoilage can deeply impact organoleptic properties [1,2,3,4,5] and color stabilization [6] but its impact is strongly wine dependent [7,8,9].

Phenolic compounds constitute primary targets to oxidation reactions [10,11,12], particularly those possessing an *ortho*-diphenol functional group which are going to be quickly converting into quinones; unstable and highly reactive compounds. Indeed, quinones are able to undergo nucleophilic addition, which is one of the main mechanisms responsible for oxidative degradation [5]. Numerous nucleophiles are available in wines, including SO_2_, phenolic compounds, amino acids, ascorbic acid, and volatile and non-volatile thiols [7,13,14,15,16,17].

Consequently, due to the manifold possible reactions and the complexity of the media, oxidation reactions effects are still difficult to predict. Thus, for example, Ferreira et al. [8] recently highlighted the unusual behavior of certain red wines towards antioxidants as SO_2_ and so the presence and competition of more reactive antioxidants.

Electrochemical methods, in particular linear sweep voltammetry and cyclic voltammetry (CV), using either carbon paste or glassy carbon electrodes, were developed and applied to the analysis of wine phenolic compounds [18,19,20,21]. Recently, electrochemical techniques became widely used to determine antioxidant capacities of food or beverages as well as to analyze polyphenols [22,23] as they appear as sensitive, fast, and easy to use techniques [24].

Cyclic voltammetry is among the most commonly used method to characterize total polyphenols content, antioxidant capacity, as well as to discriminate wine samples [25,26]. Voltammograms represent both anodic and cathodic curves. Concerning the anodic one, corresponding to the oxidation phenomenon, information can be drawn from the following parameters: (i) the peak current, proportional to the concentration of oxidizable compounds in solution; (ii) the peak potential, corresponding to the ease of oxidation of involved compounds (the more the potential is low and the more the compounds are easy to oxidize); (iii) the charge (corresponding to the area under the curve) which allows for the characterization of antioxidant capacity [22].

Glassy carbon is used as a material for classical electrochemical electrodes but presently, carbon based disposable screen-printed electrodes appear as a promising tool and are become more widely used [27]. Indeed, they offer numerous advantages, including disposability [28], reproducibility, practicality, high sensitivity, their ability to limit samples consumption thanks to miniaturization, and low detection limits [29,30]. Among these, carbon nanotubes are used, allowing for the increase in, among others, sensitivity. Two categories of carbon nanotubes electrodes exist: multi-walled carbon nanotubes (MWCNTs) and single-walled carbon nanotubes (SWCNTs) [27,31,32]. SWCNTs were privileged in this work for their good results concerning wine polyphenols characterization and antioxidant capacity as shown by Newair et al. [33]. The objectives of this article were first to determine the phenolic composition of nine different red wines (different varieties and vintage). Then, for each wine, their oxygen consumption rates (initial and average) and their voltammetry behavior with different accelerated ageing tests (H_2_O_2_, laccase and temperature) were analyzed. Finally, the possible correlations of electrochemical parameters with ageing tests results, and phenolic compounds were evaluated in order to use them as a quick method of characterization of red wine oxidation.

## 2. Materials and Methods

### 2.1. Chemicals and Reagents

Ethanol (absolute), tartaric acid (≥99.5%), laccase from *Trametes versicolor* (0.94 U·mg^−1^), Folin–Ciocalteu reagent, sodium hydroxide, chloride acid (1 M), (+)-catechin, (−)-epicatechin, quercetin and caffeic acid were purchased from Sigma-Aldrich (Saint-Quentin Fallavier, France). Oenin chloride was obtained from Extrasynthese (Genay, France).

### 2.2. Model Wine Solution

The model wine solution used as blank for electrochemical measurements was composed as follows: 12% vol. ethanol in water, 0.033 M tartaric acid and pH adjusted to 3.6 (sodium hydroxide 1 N). 

### 2.3. Red Wines

Wines with different characteristics were selected for experiments. Commercial red wines were selected from various grape varieties from four vintages, 2019, 2018, 2014 and 2010, and origins: Côtes du Rhône (Syrah 2018, 2014 and 2010 from the same winery) corresponding to R1 to R3, Chinon (Cabernet Franc, 2018) corresponding to R4, Morgon (Gamay, 2018) corresponding to R5, Alsace rouge (Pinot noir, 2018) for R6, Ardèche (Syrah, 2019) for R7, Côteaux de Béziers (Syrah 2019) for R8 and Grès-de-Montpellier (Syrah, 2019) for R9.

A bottle of each wine was opened and slowly homogenized under nitrogen to avoid oxidation reactions. Aliquots of 50 mL tubes were then immediately frozen at −80 °C.

### 2.4. Wine Global Chemical Characterization

#### 2.4.1. Wine Global Chemical Characterization

Studied wines were characterized with enological usual chemical analyses (Natoli laboratory, St Clément de Rivière, France) according to OIV procedures (www.oiv.int, accessed on 23 November 2021). Analyses included: alcoholic percentage (Fourier transformed infrared spectroscopy: FTIR); total acidity (FTIR); volatile acidity; total sulfur dioxide (automated colorimetric method); pH (FTIR); Fe (colorimetric method, reaction with disodium salt of (pyrildil-2)-3bis(phenyl-4-sulfonic 5–6 triazin-1,2,3,4) acid), absorbance read at 570 nm on a sequential analyzer (Olympus AU2700); Cu (colorimetric method 4-(3,5-Dibromo-2-Pyridilazo)-*N*-Ethyl-*N*-(3-Sulfopropyl) aniline reaction, absorbance read at 570 nm on a sequential analyzer (Olympus AU2700) (Supplementary Data Table S1).

#### 2.4.2. Quantitative Analysis of Free SO_2_ Levels (GC-MS) 

Free SO_2_ level (samples after oxygen saturation) was determinate by head-space gas chromatography coupled to a quadrupole mass spectrometer detector (HS-GC-MS) following the method described in Carrascon et al. [34] with slight modifications. GC-MS analysis was carried out on a GC Trace Ultra gas chromatograph (Thermo Fisher, Waltham, MA, USA) and coupled to an ISQ Series mass spectrometer (Thermo Fisher, Waltham, MA, USA). A DB-WAX (30 m × 0.25 mm i.d. × 0.25 µm) capillary column (Agilent Technologies, Santa Clara, CA, USA) was used for the chromatographic separation. For the analysis, 4.5 mL of sample was placed into a 10 mL headspace vial and 500 µL of *ortho*-phosphoric acid (85%) was added before closing and vials were incubated at 40 °C for 15 min. After this, 400 µL of the headspace was injected into a PTV injector, working in split mode (1:7 split ratio) and kept at 200 °C. An AOC-5000 autosampler (Shimadzu, Kyoto, Japan) with a static headspace unit was used and the 1mL gas-tight syringe was heated at 50 °C. After the injection, the hot syringe was cleaned by purging for 5 min with nitrogen. The temperature method started at 50 °C for 4 min then raised to 220 °C at 50 °C/min and was kept at this temperature for 5 min. The carrier gas employed was helium at 1.5 mL/min. The mass spectrometer was used with an electron impact (EI) ion source and acquisition was performed in single ion monitoring (SIM) mode. The m/z used for quantification were 48 and 64. 

External calibration curves in model wine containing known amounts of SO_2_ (as potassium metabisulfite) were prepared.

### 2.5. Accelerated Ageing Tests

Three accelerated oxidation tests were applied to the nine wines after oxygen saturation. Each wine sample was saturated with air by vigorously shaking a 500 mL flask containing 35 mL of wine for 10 s, after which the cap was opened (5 s) to let fresh air enter the flask. This saturation operation was repeated three times. The procedure and the test optimization have been previously described by Deshaies et al. [35]:

Briefly, enzymatic test was performed by the addition of 50 µL of a 10 mg·L^−1^ laccase from *Trametes versicolor* solution in 11 mL of red wine.

For chemical test, 20 µL of hydrogen peroxide solution (30% in water) was added to 11 mL of red wine.

Finally, the heat treatment was performed by heating 11 mL of wine at 60 °C.

For each test, red wine was put in Hermetic Pyrex 11 mL culture tubes (VWR 734–4224, Radnor, PA, USA) containing Pst3 oxygen sensors (Presens—Precision Sensing GmbH, Regensburg, Germany) with a minimum headspace.

### 2.6. Determination of Phenolic Composition

#### 2.6.1. Determination of Total Phenolic Content (TPC)

The Folin–Ciocalteu method, revised by Magalhaes et al. [36], was used to determine total phenolic content. Absorbances were measured on a multiplate reader (Spectrostar Nano—BMG Labtech) equipped with spectrophotometric detection and 96-well plates. Each well contained: 50 µL diluted wine (1/100) + 50 µL Folin Ciocalteu reagent (1:5 *v*/*v* in water) + 100 µL sodium hydroxide solution (0.35 M). Absorbance at 750 nm of formed blue complex was read after 3 min. 

The absorbance values for the wine samples were related to the calibration curve (gallic acid solution: 10 mg·L^−1^ to 200 mg·L^−1^). They reflected the total phenolic content in the considered wine. TPC was expressed in gallic acid equivalent (mg/L) and the result was multiplied by the associated dilution factor.

#### 2.6.2. Total Tannins Content (TC)

The Bate-Smith method was used to determine total tannins content (TC) [37]. To 4 mL 1/50 diluted wine (in water) in a tube was added 2 mL water and 6 mL HCl (12 N). The tube was then heated in a water bath at 100 °C for 30 min. The reference tube was identically treated without the heating step. A total of 1 mL ethanol (95%) was added to both tubes and absorbances at 550 nm were measured on spectrophotometer UV-1900 (Shimadzu, Marne-La-Vallée, France) against water under one cm of optical path. A*_550_* values were plotted on a standard curve drawn from reference oligomers. 

#### 2.6.3. Total Pigments Index (TP) 

Wine samples were diluted in HCL 1 M (1/100) in a hermetic flask. After 30 min out of light, absorbance at 520 nm was read on spectrophotometer UV-1900 (Shimadzu, Marne-La-Vallée, France) against water under 1 mm of optical path. The result was multiplied by the associated dilution factor [1,38].

#### 2.6.4. SO_2_ Bleaching Resistant Pigments Index (RP) 

To 10 mL wine in a hermetic flask, 150 µL of SO_2_ solution was added (Na_2_S_2_O_5_ in water at 200 g/L). After 30 min out of light, absorbance at 520 nm was read on spectrophotometer UV-1900 (Shimadzu, France) against water under 1 mm of optical path. The result was multiplied by the associated dilution factor [39].

### 2.7. Electrochemical Apparatus and Measurements

**Figure 1:**
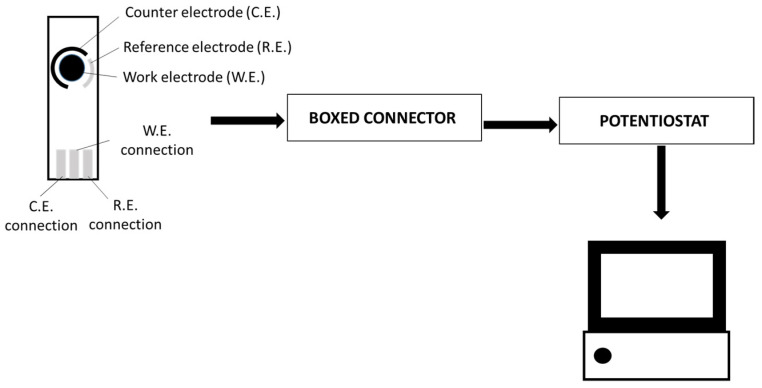
Cyclic voltammetry experimental set-up.

Electrochemical measurements were performed with potentiostat/galvanostat (Autolab PGSTAT 302N) using software Nova 2.1.5 (Metrohm, Herisau, Switzerland). Model wine solution was used to dilute wine samples (1/75). Measurements were performed in the range of −0.2 V to 0.8 V (vs. Ag_(s)_) with a scan rate of 100 mV/s using single-walled carbon nanotubes-modified screen-printed carbon electrode SWCNTs-SPCE (4 mm diameter, Dropsens, Oviedo, Spain) in a three-electrode configuration (silver solid reference electrode and carbon counter electrode). A total of 200 µL of sample were dropped at the electrode surface and measurements were immediately carried out. The experimental set-up is presented in Figure 1.

### 2.8. Statistical Analysis

The ANOVA, correlation and PCA (principal component analysis) tests were performed using XLSTAT software (Addinsoft version 19.02, Paris, France). A Tukey test was carried out and where *p*-values were <0.05 was considered as significant. Pearson’s correlation coefficient was carried out for the determination of correlations between electrochemical parameters, oxygen consumption rates and phenolic composition. All analyses, experiments and tests were performed in triplicate.

## 3. Results and Discussion

### 3.1. Wines Phenolic Characterization

According to Table 1, phenolic and tannin contents were mostly in accordance, with significant highest contents for R8 wine concerning phenolic content (3.1 g GAE·L^−1^), and R8 (4.6 g·L^−1^), R3 (4.4 g·L^−1^) and R4 (4.2 g·L^−1^) concerning total tannins content, whereas lowest contents were observed for R7 (1.4 g·L^−1^ and 2.1 g·L^−1^ respectively). These values were also in accordance with pigment index ones, with total pigment index values ranging from 4.8 for R6 to 21.2 for R9 and also the SO_2_ bleaching resistant pigments index from 1.7 to 7.2 for the same wines. Finally, it can be concluded that, although some wines were from the same cultivar, the nine wines presented very different initial phenolic composition.

### 3.2. Accelerated Ageing Tests: Oxygen Consumption Parameters

The red wines were submitted to three accelerated ageing tests experiments [36]: chemical (H_2_O_2_ added), enzymatic (by laccase from *Trametes versicolor*) and heat treatment (at 60 °C). The oxygen consumption was monitored non-destructively by means of fluorescence oxygen sensors. The wine samples submitted to ageing test oxidations consumed an oxygen concentration of approximately 6.5 mg·L^−1^ whereas, in the case of non-oxidized controls, average total consumed oxygen was only 0.20 ± 0.15 mg·L^−1^.

Table 2 data confirm that wines can differ for their kinetics to consume oxygen, in agreement with recent studies [7,8]. 

Oxidation mechanisms in wine start with oxygen activation by metal catalysis (Fe or Cu), inducing radicals named reactive oxygen species (ROS). Quinones are then formed as well as hydrogen peroxide, both capable of reacting with several other wine compounds [5,11,17]. Consequently, a decrease in the dissolved oxygen concentration is systematically observed and the measure of this reflects the evolution of wine oxidative kinetics [8,11]. 

Experiments were carried out in hermetic culture tubes with minimum headspace. It was previously shown that this procedure allowed a headspace volume between 0 µL and 120 µL [8], and so oxygen ingress through the cap was negligible and only evolution of dissolved oxygen in wine was considered during the time of the experiment. Oxygen consumption parameters are summarized in Table 2. Important differences were observed between wines both for initial and average O_2_ rates. Values ranged from 0.17 ppm·h^−1^ (R8 lac) to 4 ppm·h^−1^ (R8 H_2_O_2_) for average O_2_ rates and from 0.38 ppm·h^−1^ (R8 lac) to 9.32 ppm·h^−1^ (R8 H_2_O_2_) for initial O_2_ rates. The wines can have completely opposite behaviors towards the oxidation process. R9 and R7 wines showed similar rates around 1 ppm·h^−1^ whatever the oxidation protocol, whereas R8 and R4 wines also had low consumption rates less or around 1 ppm·h^−1^ for temperature and enzymatic tests but were among the highest rates for chemical oxidation (9.32 and 5.33 ppm·h^−1^ respectively). Globally, chemical oxidation using H_2_O_2_ showed the equal highest oxygen consumption rates for all considered samples except for R1 and R3, for which enzymatic oxidation (using laccase) showed the highest consumption rates.

Several factors can influence the oxygen consumption rates of wine as pH, ascorbic acid concentration or metals concentration [8,13] pH, and also copper and iron concentrations, are shown in the supplementary data, all play a part in oxygen consumption during experiments. For example, R8, which had the highest metals concentrations, very quickly consumed oxygen when it was oxidized adding H_2_O_2_ compared to other samples (Table 2). On the contrary, R6 had the lowest metal concentrations and was globally oxidized slower than other samples whatever the considered oxidation protocol. However, it is difficult to conclude on a clear relation between OCRs and the above variables, metals concentrations not completely reflecting the fraction involved in oxidation reactions [13,26,40,41]. 

As an antioxidant, SO_2_ can affect oxygen consumption kinetics during accelerated ageing tests. Free SO_2_ values are available in the supplementary data (Tables S1 and S2) and were measured after oxygen saturation (before oxidation) and after the different controlled oxidations. Chemical and temperature tests completely consumed available free SO_2_ (if available after saturation) whatever the initial concentration, this latter having consequently a limited impact on OCRs. This decay in SO_2_ was primarily due to its reaction with the quinones and H_2_O_2_ deriving from oxidation of phenolics [42]. Concerning enzymatic oxidation, all the available SO_2_ was not systematically consumed. However, R3 and R7 samples were statistically the ones with highest OCR_lac_ (initial and average) but these two samples presented no available free SO_2_ before enzymatic oxidation. Consequently, it can be hypothesized that free SO_2_ content had no impact on enzymatic oxidation.

Several factors can be responsible for SO_2_ consumption as ascorbic acid concentration [43] or sulfonation reactions [44]. Even if a theoretical molar reaction ratio of 2:1 (SO_2_/O_2_) should be expected [7], it is generally not observed. Recent studies showed that such a molar ratio can hardly be applied to a complex media such as wine [9,45]. Indeed, SO_2_ does not directly react with oxygen and other chemical species are involved and other strong reductants can compete with it, as recently observed by Ferreira et al. [8]. For certain wines, oxygen consumption might be really fast but not result in immediate SO_2_ loss, other highly reactive reductants can consume O_2_ with no involvement of SO_2_.

### 3.3. Electrochemical Behavior of Standard Polyphenols and Wine Samples Oxidized with the Three Different Protocols

#### 3.3.1. Electrochemical Behavior of Standard Polyphenols

**Figure 2:**
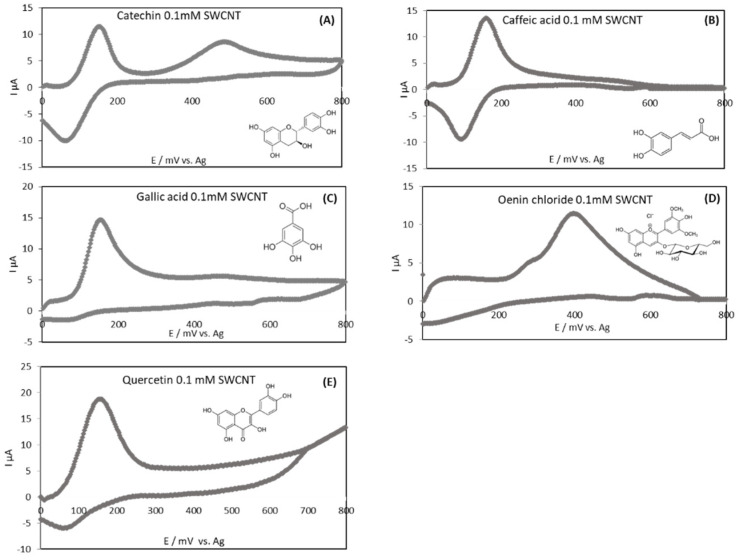
Cyclic voltammograms of different standard polyphenols at SWCNT-SCPE: catechin (**A**); caffeic acid (**B**); gallic acid (**C**); oenin chloride (**D**) and quercetin (**E**) at a concentration of 0.1 mM (blank subtracted); SWCNT-SPCE: single walled carbon nanotubes modified screen printed carbon electrodes.

Figure 2 represents the cyclic voltammograms of representative wine polyphenols (flavanol, hydroxycinnamic acid, benzoic acid, anthocyanin and flavonol) in model wine solutions using single-walled carbon nanotube (SWCNT) electrodes. The corresponding anodic (oxidation) peak potentials are given in Table 3. Only one anodic peak was present for caffeic acid at a potential of 166 mV, whereas for catechin and gallic acid two peaks were present with a potential of 151 mV for the first one (Ep,a_1_). This is attributed to the oxidation of hydroxyl groups (on B-ring for catechin) into corresponding *ortho*-quinones [19,46,47]. The formed *ortho*-quinones can be reduced on the reverse scan generating a well-defined cathodic peak as, for example, for caffeic acid (Figure 2b). The second anodic peak observed only for catechin and gallic acid (Ep,a_2_ = 476 mV) corresponded to the oxidation of catechin A-ring hydroxyl group [48] and to the oxidation of the third phenol group adjacent to the *ortho*-diphenol group in gallic acid [49]. For quercetin, only one peak at low potential (166 mV) was present and the second peak was not clearly observable. At last, concerning anthocyanins, electrochemical behavior of oenin chloride in model wine was also investigated showing only one peak at 398 mV. Oenin chloride revealed that anthocyanins tend to be less easily oxidizable compounds than other phenolic compounds.

The classification obtained considering only the first peak potential for the studied standards at equal concentration (0.1 mM) by increasing potential was as follows: quercetin 151 mV/catechin 151 mV < gallic acid 154 mV < caffeic acid 166 mV < oenin chloride 398 mV and revealed the ability of the compounds to oxidize at different potentials. These potentials depend on a molecule structure’s chemical reactivity. This ranking appears to be logical when considering substituents to catechol groups. An electro donating group reduces potential values while electro accepting groups (as carboxyl) tend to increase potential values. Moreover, it is noteworthy that the galloyl substitution of gallic acid is more oxidizable than the catechol group of caffeic acid [33].

#### 3.3.2. Electrochemical Behavior of Wines Non-Oxidized and Oxidized with Different Oxidation Protocols

Electrochemical methods, and in particular cyclic voltammetry (CV), involve mechanisms similar to those occurring in wines [20]. CV is consequently of major interest in order to study wine oxidation. It has already been applied to the study of white wine oxidation [26,50,51] and recently, carbon-based screen printed sensors turned out to be a rapid and reproducible device for the analysis of polyphenols [52] and wine components. They give access to voltammograms resulting from the current responses from a large number of polyphenols.

Values in Table 4 showed the high variability in anodic current measurements through the resulting calculated charges, depending on each wine. Wine samples were increasingly diluted (in model wine solution) from 1/100 to 1/10 and acquisitions were performed on 1/75 diluted samples. This dilution allowed for the linear part of the total charge response as a function of wine concentration. Moreover, experiments were performed on wines diluted 75 times in order to discard free SO_2_ influence in anodic reactions (oxidation) [53]. Indeed, with higher SO_2_ contents, semi quinone radicals formed during electrochemical oxidation can react, leading to quinone reduction back to the original polyphenol [54]. Further reactions could also take place as the formation of sulfonic acid derivatives, disrupting CV results by increasing anodic peak intensity and also decreasing the cathodic peak intensity [54]. 

**Figure 3:**
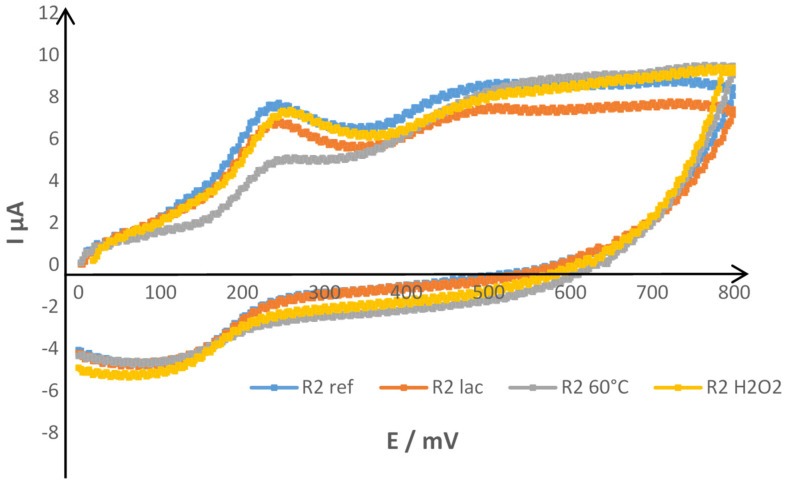
Cyclic voltammograms of R2 wine non-oxidized (ref) and oxidized with three different protocols: temperature (60 °C), chemical (H_2_O_2_) and enzymatic (laccase) with SWCNT.

Voltammetric experiments were carried out for all reference wines as well as all oxidation protocols. Cyclic voltammograms showed for each sample three peaks at approximately the same potentials: around 220, 480 and 700 mV. Reverse cathodic peak was also observed. An example of obtained cyclic voltammograms for the reference R2 wine and the three accelerated protocols (blank subtracted) is presented in Figure 3. 

The first oxidation peak can be assigned to the most oxidizable compounds as catechin-type flavonoids [52], including oligomers and polymers, caffeic acid and derivatives, flavonols containing a catechol or a galloyl group (Table 3). As described by Newair et al. [33], red wines contain high concentrations of flavan-3-ols, caffeic acid and gallic acid, all responsible for the first anodic peak intensity. 

The second peak on the voltammograms at around 520 mV could correspond to overlaps [53] mainly resulting from the malvidin anthocyanins oxidation as well as to the second oxidation of catechin type flavanols and galloyl containing compounds (Table 3). Ferulic acid and trans resveratrol or similar polyphenols could also interact [18].

Concerning the third peak, its presence was less marked on the voltammograms and can corresponds to the oxidation of phenolic compounds with high oxidation potentials, such as para-coumaric or vanillic acids [19,55,56]. The presence of a cathodic peak on the reverse scan on the voltammograms is noteworthy.

As mentioned previously, differences between wines were mainly notables on intensities on cyclic voltammograms. In addition to that, oxidized wine intensities on voltammograms were almost equal or inferior to the reference ones corresponding, reflecting the oxidation protocol impact on the oxidizable compound content in the sample. For example, concerning the R2 wine in Figure 3 representing cyclic voltammograms for reference wine and three accelerated protocols, enzymatic and chemical oxidation protocols appeared to have a moderate impact on the wine compared to temperature oxidation, with the latter having the most important one. 

#### 3.3.3. Charges of Non-Oxidized Red Wines and Charge Variation after Accelerated Ageing Tests

The different charges extracted from voltammograms and corresponding to the area under the anodic curve until the given potential value shown in Table 4 allow us to illustrate the polyphenol’s capacity to be oxidized at the working electrode [52]. The total charge Q_800mV_ corresponds to all oxidizable phenolic compounds that will contribute to the total antioxidant capacity of the wine sample. Q_240mV_ represents the electrochemical charge of the most easily oxidizable polyphenols (generally hydroxyl group oxidation into quinones for flavan-3-ols, phenol acids and flavonols) that have consequently the highest antioxidant capacity. Q_520mV_ estimates the charge of the compounds which oxidize until 520 mV (the major part). In addition to that, the difference (Q_520mV_–Q_240mV_) gives the charge of the compounds that correspond to the second oxidation peak on the voltammograms, whereas the ratio Q_240mV_/Q_800mV_ illustrates the contribution of the most antioxidant compounds to the total antioxidant capacity of the wine.

For the reference wines (Table 4A), the charges (Q) values were extracted from voltammograms with blank subtracted (model wine solution), whereas for the oxidized wine samples (Table 4B–D), the difference of charges (ΔQ) values were extracted from voltammograms corresponding to oxidized wines with reference wine subtracted in order to obtain values representing only oxidized compounds.

For non-oxidized wines the obtained charges values (Q_240mV_, Q_520mV_ and Q_800mV_) were very different (Table 4A). The lowest values for the charges were observed for R7 wine and these weak values were in accordance with its phenolic and total tannins contents which were also the lowest ones (Table 1). On the opposite end, the highest values were observed for R8 wine which presents a significantly high content of polyphenols and tannins, compared to other wines.

Values in Table 4B–D were those of reference wines minus oxidized wines (ΔQ) in order to take into consideration only oxidation effect. As described by previous researchers [18,19,20], the majority of compounds involved in wine oxidation reactions (phenolic compounds, ascorbic acid) are able to oxidize at the surface of an electrode and therefore can be analyzed using voltammetric methods. A comparison of oxidized wines with their corresponding non-oxidized ones (subtractive approach) can provide additional information on global wine oxidation. Indeed, due to the numerous simultaneous voltammetric signals occurring during wine analysis, this method is unable to provide specific information on individual wine components.

Interestingly, charge differences (ΔQ) of subtractive voltammograms were also strongly wine-dependent, which provides interesting information on chemical reactions involving the major wine oxidizable compounds during oxygen consumption. Some wines showed important current decrease at 240 mV and consequently high values of difference of charge. It was the case for R8 wine oxidized with laccases or at 60 °C or R6 wine for all oxidation types. It revealed that they contained oxidizable species which were easily degraded upon oxygen consumption. On the other hand, other wines showed opposite behavior, as for R7 wine oxidized with H_2_O_2_ or at 60 °C. Moreover, the different values of Δ(Q_520mV_–Q_240mV_) indicated that oxidation also induced changes in less easily oxidizable compounds.

All these measurements were also very different depending on the considered oxidation protocol, as for R2 wine values, which were among the highest ones with temperature oxidation and among the lowest ones with laccase or H_2_O_2_ oxidation protocols, whereas R1 wine showed opposite trends for the same protocols. These results are in accordance with the influence of wine composition on its oxidation ability.

It is noteworthy that even older wines (R2 and R3 were respectively from 2014 and 2010) contain oxidizable compounds and can undergo further oxidations. Hydrogen peroxide had an impact on them whereas R7 and R8 wines were very little impacted. On the contrary, enzymatic oxidation had a negligible impact on them. Temperature oxidation impacts R2 and R3 wines whereas it was not true for R1 wine (2018 Syrah wine). Even if oxidation phenomena are strongly wine dependent, these results can suggest that oxidation reactions lead to the formation of different products which are able to further oxidize themselves.

### 3.4. Correlation between Phenolic Composition, Oxygen Consumption Rates and Electrochemical Parameters

Table 5 represents the Pearson correlation matrix between electrochemical parameters for non-oxidized wines and for all accelerated ageing tests (H_2_O_2_, laccase and at 60 °C), OCRs (initial and average oxygen consumption rate) and wine composition (tannins, polyphenols, total pigments, and SO_2_ bleaching resistant pigments).

Concerning the phenolic composition, the charges Q_240mV ref_ and Q_520mV ref_ were well correlated with TPC (itself well correlated with TC (R = 0.91)) with R = 0.86 and 0.80 respectively. A better correlation between TPC and the total charge Q_800mV ref_ could have been expected since the Folin–Ciocalteu test (TPC) is a global method for which all hydroxyl groups that are able to react with the F-C reagent are implied [34]. These three charges were also well correlated with the values of their corresponding difference of charge at the same potential for enzymatic and temperature oxidation tests, while no correlation was observed with chemical oxidation using hydrogen peroxide. The fact that the calculated charges of non-oxidized (reference) wines, in accordance with its oxidability, presented high correlations with the difference of the charges for enzymatic as well as temperature oxidized wines (reference subtracted) emphasizes that cyclic voltammetry can partly predict these two oxidation tests and reflects the wine sensitivity towards respective oxidation targets. However, it is not possible to predict the wine chemical oxidation test using hydrogen peroxide. These last results are in accordance with the good negative correlations between the oxygen consumption rates and the charges of the reference wines: between aOCR_lac_ and Q_240mV ref_ (R = −0.82) or Q_800mV ref_ (R = −0.84), and between iOCR_60°C_ and Q_240mV ref_, Q_520mV ref_ and Q_800mV ref_ (R = −0.84; −0.82 and −0.88 respectively).

Considering the parameters related to oxygen consumption kinetics, for a same oxidation test, iOCR was highly correlated with aOCR: R(iOCR_60°C_/aOCR_60°C_) = 0.89; R(iOCR_lac_/aOCR_lac_) = 0.95 and R(iOCR_H2O2_/aOCR_H2O2_) = 0.90. These results are in accordance with previous studies studying OCR of oxygen saturated white wines [26]. It is worth mentioning that OCRs for enzymatic by laccase and temperature at 60 °C oxidations were partly correlated to each other (iOCR_60°C_ correlated with aOCR_lac_ and iOCR_lac_ with respectively R = 0.85 and 0.80).

Concerning the relation between OCRs and the differences of charges (ΔQ) for oxidized wines, strong negative correlations were observed for the temperature oxidation between the oxygen consumption rates and the charge at low potential ΔQ_240mV 60°C_ as well as the total charge ΔQ_800mV 60°C_ and more particularly for iOCR_60°C_. For the laccase oxidation test, less robust correlations were obtained.

Kinetic parameters for the H_2_O_2_ ageing test (iOCR_H2O2_ or aOCR_H2O2_) were not correlated with the electrochemical parameters of reference wines but showed a high negative correlation with ΔQ_520mV H2O2_ with R = −0.88 and −0.84, respectively (R = −0.84 between aOCR_H2O2_ and ΔQ_520mV H2O2_).

### 3.5. Principal Component Analysis

**Figure 4:**
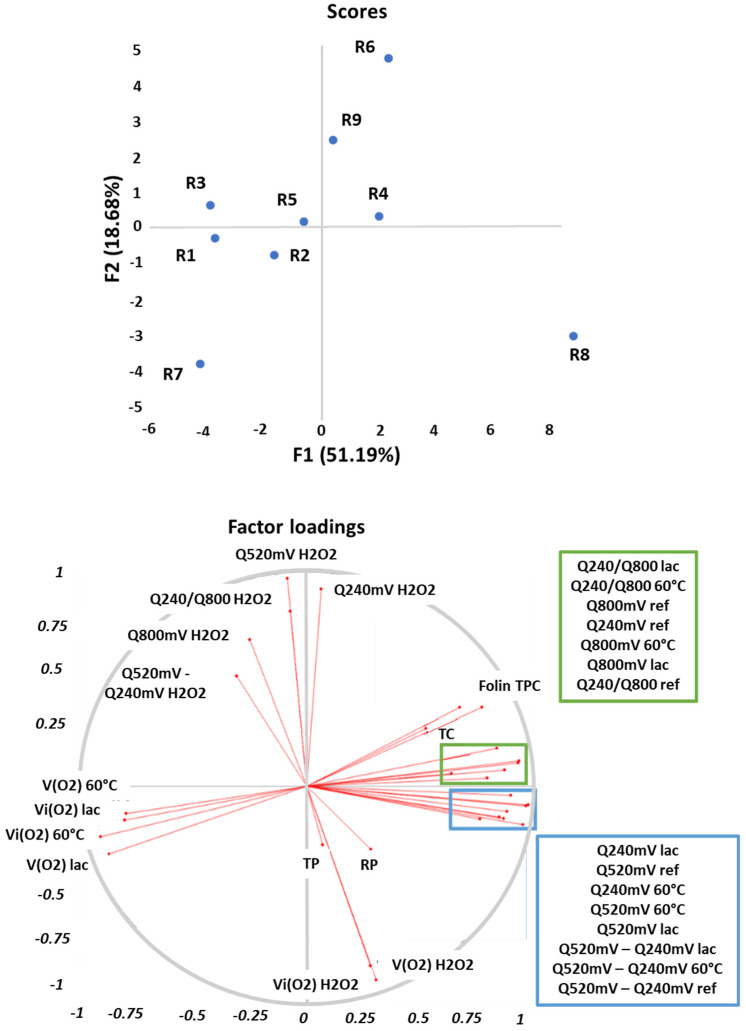
Representation of the loadings (variables) and the scores (wines) in the plane defined by respectively the first (F1) and second (F2) factor (explained variance: 69.87%).

Principal component analysis (PCA) was used in this work in order to classify wines. Factor analysis was calculated with the same parameters as the ones used for Pearson’s correlation in Table 5. Figure 4 shows the plots of loadings and scores in the space described by the 1st and 2nd factors. 

The first factor explains 51.19% of the total data variability and it is mainly related to the richness in polyphenols and tannins (TPC and TC) and to the electrochemical parameters for non-oxidized wines, enzymatic and temperature oxidations, and their respective OCRs. It allows us to distinguish red wine samples depending on their oxidation rates with laccases or at 60 °C. Indeed, the first factor is positively correlated with electrochemical parameters, TPC and TC, while it is negatively correlated with OCRs. R1, R3, as well as R7, are red wines being the fastest oxidized by laccases and by temperature. R1 and R7 wines are among the lowest in TPC and TC values.

The second factor explains 18.68% of the total data variability. It is positively correlated with electrochemical parameters concerning chemical oxidation with H_2_O_2_ and negatively correlated with corresponding OCRs and with anthocyanins content (TP and RP). R8 is the red wine with the highest TPC and TC and the fastest oxidized by hydrogen peroxide, and the slowest oxidized by the two other tests.

Wines can consequently be discriminated in Figure 4 into three groups depending on their variable responses. The three Syrah wines (R1, R2 and R3) from the same winery but with different vintages are gathered together on the left of the PCA while both 2019 aged Syrah wines (R7 and R8) are at the bottom of Figure 4. All other wines from different grape cultivars are gathered in a third group, also containing the last 2019 aged Syrah wine (R9), a hypothesis being that it is the only Syrah wine in the study which was aged in barrels.

**Figure 5:**
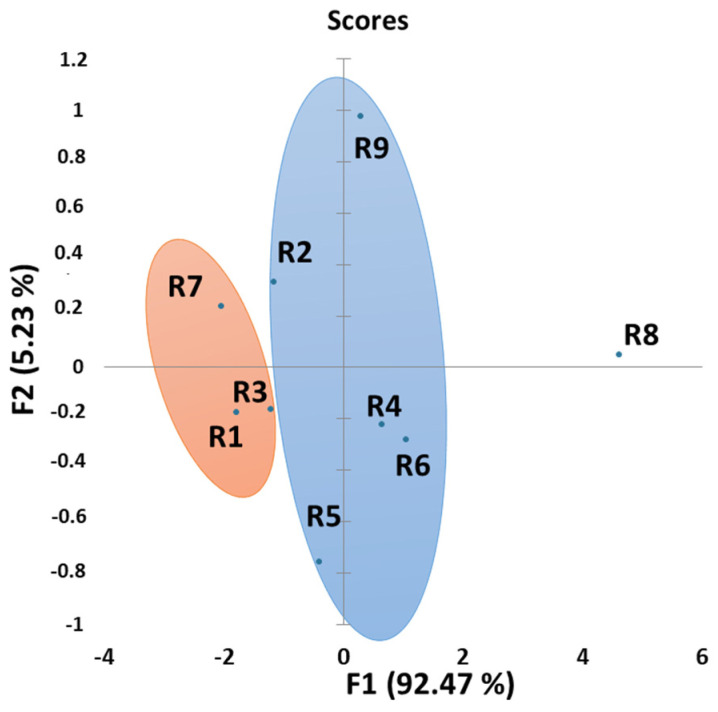

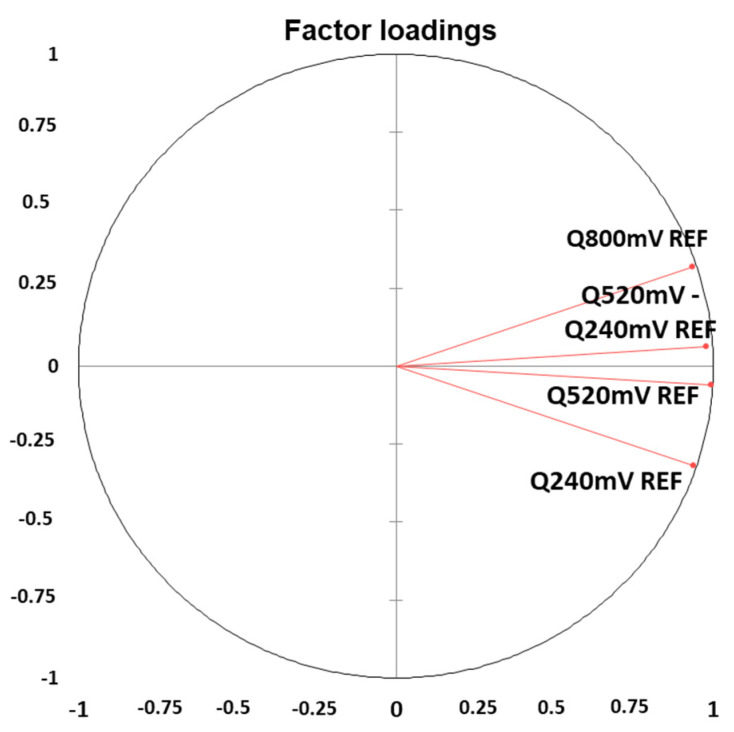
Representation of the loadings (variables—electrochemical parameters (except Q_240mV_/Q_800mV_ ratio) for the reference wines) and the scores (wines) in the plane defined by respectively the first (F1) and second (F2) factor (explained variance: 97.74%).

Figure 5 is a PCA of the reference wines according to their electrochemical parameters (except Q_240mV_/Q_800mV_ ratio). It shows the plots of loadings and scores in the space described by the 1st and 2nd factors. The first factor explains 92.47% of the total data variability while the second one explains 5.23%. Three groups can be seen on Figure 5 depending on wine OCRs (initial or average). Indeed, the first group contains R1, R3 and R7 wines, and corresponds to the wines which consume oxygen the most quickly when oxidized with temperature or laccases, while R8 wine shows really low OCRs with these two oxidation processes. All other wines (R2, R4, R5, R6 and R9) have intermediate values compared the two previous groups. Consequently, electrochemical measurements on non-oxidized (reference) wines allow us to classify wines depending on their OCRs (for laccase and temperature). It is then possible to predict a wine’s sensibility towards oxidation (laccase and temperature) thanks to electrochemical measurements of the reference wines. This method is very promising as it is quick and necessitates a sample diluted drop.

Another PCA representation gathering all the electrochemical parameters (before and after oxidation) also reveals a discrimination into three main groups (Supplementary data Figure S1) and confirms previous results.

## 4. Conclusions

Overall, the results of this work confirm that different wine ageing tests produce distinct results for different red wines, depending on the chemical molecules targeted [8,34,57]. These wines also showed different electrochemical behavior (cyclic voltammetry) before and after oxidation protocols. Wines were discriminated into three main groups depending on their electrochemical behavior, chemical composition, and OCRs: the first group gathered three Syrah wines from the same winery with different ages, the second one gathered two 2019 Syrah wines, while the other samples were gathered in the last group.

Electrochemical parameters before oxidation (temperature and laccases) revealed a discrimination into three groups between red wine samples according to their oxygen consumption rates. Electrochemistry and more specifically cyclic voltammetry appears then as a promising method to predict red wine oxidation and seems to be effective even on older vintages. Moreover, this method appears to be fast and allows us to obtain results with really few samples.

Further research is needed in order to confirm the obtained results by the oxidative and electrochemical characterization in a larger series of red wines.

## Figures and Tables

**Table 1:** Phenolic characterization of the different red wines. Values represent means of triplicate determination ± SD. GAE: gallic acid equivalent. Different letters indicate the significant differences between samples in one column according to Tukey’s test, *p* < 0.05.

Wine Sample	Total Phenolic Content (g GAE/L)	Total Tannins Content (g/L)	Total Pigments Index	SO_2_ Bleaching Resistant Pigments Index
R1	1.9 ± 0.1 ^d^	2.8 ± 0.2 ^d^	19.2 ± 0.9 ^a^	5.4 ± 0.4 ^b^
R2	1.9 ± 0.1 ^d^	3.2 ± 0.1 ^c,d^	4.9 ± 0.1 ^d^	4.3 ± 0.1 ^c,d^
R3	2.5 ± 0.1 ^b^	4.4 ± 0.1 ^a,b^	6.0 ± 0.5 ^d^	4.8 ± 0.1 ^b,c^
R4	2.4 ± 0.1 ^b^	4.2 ± 0.1 ^a,b^	8.35 ± 1.1 ^c^	4.6 ± 0.3 ^b,c^
R5	2.6 ± 0.1 ^b,c^	3.4 ± 0.1 ^c^	9.1 ± 0.1 ^c^	3.5 ± 0.2 ^d^
R6	2.7 ± 0.2 ^b^	2.8 ± 0.1 ^d^	4.8 ± 0.3 ^d^	1.7 ± 0.1 ^e^
R7	1.4 ± 0.1 ^e^	2.1 ± 0.1 ^e^	14.7 ± 0.5 ^b^	3.5 ± 0.4 ^d^
R8	3.1 ± 0.05 ^a^	4.6 ± 0.1 ^a^	16.2 ± 1.3 ^b^	6.8 ± 0.2 ^a^
R9	2.5 ± 0.1 ^b^	4.1 ± 0.2 ^b^	21.2 ± 1.9 ^a^	7.2 ± 0.1 ^a^

**Table 2:** Oxygen consumption rates (ppm·h^−1^) of wines for the accelerated ageing tests. Values represent means of triplicate determination ± SD. Initial O_2_ rate (iOCR) is calculated as tangent to the linear regression O_2_ = f (time) in the first 30 min. Average O_2_ rate (aOCR) is calculated between the first O_2_ value before the aging test and the first O_2_ value of the final threshold. Different letters indicate the significant differences between samples in one column according to Tukey’s test, *p* < 0.05.

	Initial O_2_ Rate iOCR	Average O_2_ Rate aOCR		Initial O_2_ Rate iOCR	Average O_2_ Rate aOCR		Initial O_2_ Rate iOCR	Average O_2_ Rate aOCR
R1 60 °C	2.40 ± 0.06 ^a,b^	1.27 ± 0.05 ^a^	R1 lac	3.33 ± 0.05 ^b,c^	1.35 ± 0.21 ^a,b^	R1 H_2_O_2_	2.86 ± 0.28 ^c,d^	1.44 ± 0.21 ^c^
R2 60 °C	1.83 ± 0.52 ^a,b,c^	0.71 ± 0.30 ^b,c,d^	R2 lac	2.58 ± 0.40 ^c,d^	1.19 ± 0.13 ^b,c^	R2 H_2_O_2_	5.1 ± 0.19 ^b^	2.65 ± 0.74 ^b^
R3 60 °C	2.59 ± 0.32 ^a^	1.02 ± 0.10 ^a,b^	R3 lac	5.47 ± 0.82 ^a^	1.9 ± 0.50 ^a^	R3 H_2_O_2_	3.74 ± 0.05 ^b,c^	2.35 ± 0.17 ^b,c^
R4 60 °C	1.07 ± 0.09 ^c,d^	0.54 ± 0.11 ^c,d^	R4 lac	1.2 ± 0.04 ^e,f^	0.77 ± 0.09 ^b,c,d^	R4 H_2_O_2_	5.33 ± 0.77 ^b^	2.9 ± 0.12 ^b^
R5 60 °C	2.14 ± 0.10 ^a,b^	0.93 ± 0.11 ^a,b,c^	R5 lac	1.4 ± 0.05 ^e,f^	0.8 ± 0.05 ^b,c,d^	R5 H_2_O_2_	4.89 ± 0.89 ^b^	2.4 ± 0.14 ^b,c^
R6 60 °C	0.87 ± 0.21 ^c,d^	0.33 ± 0.12 ^d^	R6 lac	1.85 ± 0.11 ^d,e^	0.57 ± 0.13 ^c,d^	R6 H_2_O_2_	1.69 ± 0.58 ^d^	0.25 ± 0.08 ^d^
R7 60 °C	2.4 ± 0.24 ^a,b^	0.86 ± 0.02 ^a,b,c^	R7 lac	4.2 ± 0.09 ^b^	2 ± 0.08 ^a^	R7 H_2_O_2_	8.55 ± 0.19 ^a^	2.72 ± 0.09 ^b^
R8 60 °C	0.7 ± 0.11 ^d^	0.37 ± 0.06 ^d^	R8 lac	0.38 ± 0.10 ^f^	0.17 ± 0.03 ^d^	R8 H_2_O_2_	9.32 ± 0.25 ^a^	4 ± 0.15 ^a^
R9 60 °C	1.61 ± 0.43 ^b,c,d^	0.89 ± 0.01 ^a,b,c^	R9 lac	1 ± 0.05 ^e,f^	0.54 ± 0.01 ^c,d^	R9 H_2_O_2_	1.69 ± 0.05 ^d^	0.22 ± 0.06 ^d^

**Table 3:** Voltammetric peak potentials of the standard polyphenols (concentration of 0.1 mM) in model wine solution (pH 3.6) using SWCNT-SPCE. Ep,a_1_ represents the potential of the first anodic peak and Ep,a_2_ represents the potential of the second anodic peak.

Standards	Potential (mV) SWCNT-SPCE (vs. Ag_(s)_)	
	Ep,a_1_	Ep,a_2_
Catechin	151	476
Caffeic acid	166	/
Gallic acid	154	476
Oenin chloride	398	/
Quercetin	151	/

**Table 4:** Charges (Q) for reference (non-oxidized) wines (blanck substracted) and difference of charges (ΔQ) between reference wines and oxidized wines with three different accelerated protocols (60 °C, laccase and H_2_O_2_). Q and ΔQ are expressed in µC. Different letters indicate the significant differences between samples in one column according to Tukey’s test, *p* < 0.05.

A	**Q_240mV_**		**Q_520mV_**	**Q_520mV_–Q_240mV_**	**Q_240mV_/Q_800mV_**	**Q_800mV_**
R1	7.64 ± 0.76 ^d^		20.49 ± 1.74 ^d^	12.83 ± 1.12 ^e^	0.21 ± 0.03 ^b,c^	37.30 ± 1.98 ^e^
R2	7.42 ± 1.26 ^d^		26.11 ± 2.23 ^d^	18.54 ± 1.01 ^d,e^	0.15 ± 0.02 ^c,d^	50.25 ± 3.21 ^c,d,e^
R3	9.67 ± 1.55 ^c,d^		27.62 ± 3.32 ^d^	17.94 ± 1.67 ^d,e^	0.23 ± 0.06 ^b,c^	42.57 ± 3.87 ^e^
R4	19.00 ± 5.41 ^b^		46.00 ± 3.02 ^b,c^	27.96 ± 2.21 ^b,c^	0.29 ± 0.03 ^a,b^	65.11 ± 12.92 ^b,c,d^
R5	16.21 ± 1.93 ^b,c^		38.25 ± 0.29 ^c^	22.04 ± 1.42 ^c,d^	0.38 ± 0.01 ^a^	43.29 ± 6.78 ^d,e^
R6	20.73 ± 0.46 ^b^		51.30 ± 4.36 ^b^	30.55 ± 3.62 ^b^	0.31 ± 0.01 ^a,b^	67.75 ± 0.96 ^b,c^
R7	3.60 ± 0.74 ^d^		17.93 ± 1.82 ^d^	14.33 ± 1.07 ^e^	0.09 ± 0.01 ^d^	37.75 ± 6.28 ^e^
R8	29.28 ± 2.77 ^a^		96.52 ± 1.12 ^a^	69.71 ± 0.87 ^a^	0.30 ± 0.03 ^a,b^	99.00 ± 1.88 ^a^
R9	10.67 ± 2.13 ^c,d^		38.98 ± 4.40 ^c^	28.31 ± 2.25 ^b,c^	0.14 ± 0.04 ^c,d^	75.98 ± 8.51 ^a,b^
B		**ΔQ_240mV_**	**ΔQ_520mV_**	**Δ(Q_520mV_–Q_240mV_)**	**Δ(Q_240mV_/Q_800mV_)**	**ΔQ_800mV_**
R1	60 °C	0 ^f^	0 ^d^	0 ^f^	0 ^a^	0 ^d^
R2	60 °C	8.20 ± 0.86 ^b,c^	17.30 ± 0.87 ^c^	9.11 ± 0.01 ^d^	0.34 ± 0.03 ^a^	24.11 ± 0.10 ^b^
R3	60 °C	0.56 ± 0.53 ^e,f^	3.88 ± 0.46 ^d^	3.35 ± 0.07 ^e^	0.05 ± 0.05 ^a^	10.62 ± 0.8 ^c,d^
R4	60 °C	10.70 ± 0.37 ^b^	70.20 ± 0.56 ^a^	59.53 ± 0.19 ^a^	0.24 ± 0.01 ^a^	44.32 ± 0.50 ^a^
R5	60 °C	3.36 ± 2.05 ^d,e^	5.50 ± 4.91 ^d^	2.13 ± 2.87 ^e,f^	0.52 ± 0.60 ^a^	11.70 ± 7.22 ^c^
R6	60 °C	10.56 ± 1.06 ^b^	23.80 ± 1.64 ^b^	13.24 ± 0.58 ^c^	0.40 ± 0.03 ^a^	26.42 ± 4.41 ^b^
R7	60 °C	0 ^f^	2.35 ± 1.09 ^d^	2.34 ± 1.02 ^e,f^	0 ^a^	4.61 ± 1.12 ^c,d^
R8	60 °C	23.22 ± 1.66 ^a^	49.04 ± 6.16 ^a^	50.23 ± 0.82 ^b^	0.48 ± 0.03 ^a^	73.45 ± 2.48 ^a^
R9	60 °C	5.64 ± 1.06 ^c,d^	16.27 ± 2.15 ^c^	10.64 ± 1.09 ^c,d^	0.17 ± 0.02 ^a^	31.98 ± 5.26 ^b^
C		**ΔQ_240mV_**	**ΔQ_520mV_**	**Δ(Q_520mV_–Q_240mV_)**	**Δ(Q_240mV_/Q_800mV_)**	**ΔQ_800mV_**
R1	Lac.	3.37 ± 0.60 ^c,d,e^	5.74 ± 1.59 ^d,e^	2.36 ± 1.00 ^d,e^	0.30 ± 0.09 ^a,b^	11.37 ± 1.48 ^c^
R2	Lac.	0.22 ± 0.17 ^e^	0.61 ± 0.24 ^e,f^	0.40 ± 0.08 ^e^	0.02 ± 0.01 ^c^	9.49 ± 2.47 ^c^
R3	Lac.	0 ^e^	0 ^f^	0 ^e^	0 ^c^	1.61 ± 0.53 ^c^
R4	Lac.	2.05 ± 0.13 ^d,e^	1.48 ± 0.52 ^e,f^	0 ^e^	0.47 ± 0.19 ^a^	4.93 ± 2.11 ^c^
R5	Lac.	5.88 ± 1.69 ^c^	8.92 ± 2.63 ^d^	3.04 ± 0.94 ^d,e^	0.41 ± 0.03 ^a^	14.15 ± 3.04 ^c^
R6	Lac.	11.80 ± 1.29 ^b^	32.31 ± 1.48 ^b^	20.50 ± 0.22 ^b^	0.40 ± 0.02 ^a^	29.13 ± 1.43 ^b^
R7	Lac.	0.63 ± 0.51 ^e^	4.68 ± 1.63 ^d,e,f^	4.03 ± 1.12 ^d^	0.05 ± 0.03 ^c^	12.18 ± 4.83 ^c^
R8	Lac.	22.80 ± 2.31 ^a^	46.31 ± 8.55 ^a^	48.78 ± 1.59 ^a^	0.50 ± 0.04 ^a^	71.58 ± 0.72 ^a^
R9	Lac.	5.70 ± 2.09 ^c,d^	16.43 ± 4.40 ^c^	10.73 ± 2.31 ^c^	0.18 ± 0.02 ^b,c^	31.49 ± 8.20 ^b^
D		**ΔQ_240mV_**	**ΔQ_520mV_**	**Δ(Q_520mV_–Q_240mV_)**	**Δ(Q_240mV_/Q_800mV_)**	**ΔQ_800mV_**
R1	H_2_O_2_	3.29 ± 0.13 ^c,d^	5.73 ± 0.08 ^c,d,e^	2.45 ± 0.05 ^b,c^	0.07 ± 0.01 ^a^	45.39 ± 2.64 ^a^
R2	H_2_O_2_	1.45 ± 0.04 ^d,e^	4.05 ± 1.08 ^d,e,f^	2.7 ± 0.88 ^b^	0.55 ± 0.58 ^a^	5.28 ± 4.12 ^c,d^
R3	H_2_O_2_	3.66 ± 0.88 ^b,c^	9.56 ± 1.13 ^b,c^	5.87 ± 0.25 ^a^	0.18 ± 0.03 ^a^	19.77 ± 1.81 ^b^
R4	H_2_O_2_	2.22 ± 0.32 ^c,d^	8.13 ± 0.56 ^b,c,d^	5.91 ± 0.23 ^a^	0.16 ± 0.08 ^a^	17.22 ± 9.48 ^b,c^
R5	H_2_O_2_	2.68 ± 0.89 ^c,d^	5.36 ± 2.49 ^c,d,e^	2.68 ± 1.57 ^b^	0.11 ± 0.03 ^a^	23.75 ± 0.53 ^b^
R6	H_2_O_2_	13.11 ± 1.46 ^a^	15.50 ± 3.31 ^a^	2.39 ± 1.85 ^b,c^	0.30 ± 0.01 ^a^	43.27 ± 6.75 ^a^
R7	H_2_O_2_	0 ^e^	1.35 ± 0.19 ^e,f^	1.35 ± 0.19 ^b,c^	0 ^a^	3.77 ± 1.45 ^c,d^
R8	H_2_O_2_	0 ^e^	0 ^f^	0 ^c^	0 ^a^	0 ^d^
R9	H_2_O_2_	5.72 ± 1.03 ^b^	11.92 ± 1.90 ^a,b^	6.20 ± 0.87 ^a^	0.39 ± 0.09 ^a^	15.59 ± 5.98 ^b,c^

**Table 5:** Pearson’s correlation coefficients between electrochemical parameters, phenolic composition, and oxygen consumption rates. * (in green) represents significance at *p* ≤ 0.05 and ** (in red) represent significance at *p* ≤ 0.01.

	**Folin TPC**	**TC**	**TP**	**RP**	**Q_240mV ref_**	**Q_520mV ref_**	**Q_520mV_–Q_240mV ref_**		**Q_240/800mV ref_**	**Q_800mV ref_**	**ΔQ_240mV H2O2_**	**ΔQ_520mV H2O2_**	**Δ(Q_520mV_–Q_240mV_)_H2O2_**		**Q_240/800mV H2O2_**	**ΔQ_800mV H2O2_**
**Folin TPC**	**1.00**	0.91 **	−0.08	0.26	0.86 **	0.80 **	0.74 *		0.53	0.74 *	0.31	0.29	0.09		0.00	0.06
**TC**		**1.00**	−0.15	0.36	0.72 *	0.69 *	0.65		0.33	0.73 *	0.25	0.36	0.37		0.14	−0.07
**TP**			**1.00**	0.72 *	−0.10	0.09	0.18		−0.49	0.21	−0.26	−0.25	−0.09		−0.35	−0.06
**RP**				**1.00**	0.10	0.32	0.41		−0.40	0.45	−0.45	−0.29	0.18		−0.06	−0.38
**Q_240mV ref_**					**1.00**	0.94 **	0.87 **		0.66	0.80 **	0.18	0.03	−0.25		−0.23	−0.03
**Q_520mV ref_**						**1.00**	0.99 **		0.43	0.90 **	0.00	−0.16	−0.35		−0.23	−0.27
**Q_520mV_–Q_240mV ref_**							**1.00**		0.31	0.90 **	−0.08	−0.24	−0.39		−0.23	−0.37
**Q_240/800mV ref_**									**1.00**	0.09	0.14	0.00	−0.25		−0.35	0.31
**Q_800mV ref_**										**1.00**	0.14	0.07	−0.09		0.07	−0.30
**ΔQ_240mV H2O2_**											**1.00**	0.90 **	0.21		0.37	0.70 *
**ΔQ_520mV H2O2_**												**1.00**	0.62		0.47	0.63
**Δ(Q_520mV_–Q_240mV_)_H2O2_**													**1.00**		0.39	0.14
**ΔQ_240/800mV H2O2_**															**1.00**	0.00
**ΔQ_800mV H2O2_**																**1.00**
	**ΔQ_240mV lac_**	**ΔQ_520mV lac_**	**Δ(Q_520mV_-Q_240mV_)_lac_**	**ΔQ_240/800mV lac_**	**ΔQ_800mV lac_**	**ΔQ_240mV 60°C_**	**ΔQ_520mV 60°C_**	**Δ(Q_520mV_–Q_240mV_)_60°C_**	**ΔQ_240/800mV 60°C_**	**ΔQ_800mV 60°C_**	**aOCR H_2_O_2_**	**aOCR lac**	**aOCR 60 °C**	**iOCR H_2_O_2_**	**iOCR lac**	**iOCR 60 °C**
**Folin TPC**	0.71 *	0.65	0.53	0.62	0.58	0.67 *	0.55	0.49	0.66	0.65	0.02	−0.77 *	−0.49	−0.12	−0.58	−0.63
**TC**	0.49	0.48	0.33	0.40	0.39	0.63	0.63	0.60	0.43	0.74 *	0.06	−0.61	−0.48	−0.15	−0.43	−0.60
**TP**	0.23	0.26	−0.49	0.10	0.43	−0.03	−0.03	−0.02	−0.35	−0.03	−0.10	−0.15	0.36	0.08	−0.22	0.07
**RP**	0.24	0.29	−0.40	0.02	0.36	0.26	0.29	0.28	−0.15	0.35	0.23	−0.27	0.21	0.14	−0.27	−0.06
**Q_240mV ref_**	0.86 **	0.81 **	0.66	0.83 **	0.65	0.87 **	0.80 **	0.73 *	0.74 *	0.76 *	0.25	−0.82 **	−0.75 *	0.21	−0.71 *	−0.84 **
**Q_520mV ref_**	0,93 **	0.92 **	0.43	0.69 *	0.79 *	0.94 **	0.81 **	0.72 *	0.66	0.80 **	0.36	−0.79 *	−0.72 *	0.38	−0.69 *	−0.82 **
**Q_520mV_–Q_240mV ref_**	0.93 **	0.94 **	0.31	0.59	0.83 **	0.93 **	0.78 *	0.68 *	0.60	0.78 *	0.40	−0.74 *	−0.68 *	0.44	−0.65	−0.77 *
**Q_240/800mV ref_**	0.39	0.28	**1.00** **	0.68 *	0.02	0.34	0.34	0.32	0.62	0.16	0.28	−0.33	−0.33	0.11	−0.25	−0.31
**Q_800mV ref_**	0.81 **	0.82 **	0.09	0.54	0.83 **	0.90 **	0.79 *	0.71 *	0.53	0.91 **	0.10	−0.84 **	−0.74 *	0.16	−0.76 *	−0.88 **
**ΔQ_240mV H2O2_**	0.13	0.08	0.14	0.17	0.20	−0.02	−0.16	−0.20	0.15	0.02	−0.84 **	−0.28	−0.27	−0.77 *	−0.10	−0.31
**ΔQ_520mV H2O2_**	−0.14	−0.19	0.00	0.04	−0.04	−0.18	−0.16	−0.15	−0.03	0.05	−0.84 **	−0.17	−0.10	−0.88 **	−0.02	−0.17
**Δ(Q_520mV_–Q_240mV_)_H2O2_**	−0.55	−0.57	−0.25	−0.23	−0.45	−0.35	−0.08	0.02	−0.34	0.07	−0.36	0.12	0.27	−0.59	0.14	0.17
**ΔQ_240/800mV H2O2_**	−0.28	−0.28	−0.35	−0.38	−0.08	−0.01	−0.14	−0.18	0.16	0.17	−0.44	−0.14	−0.12	−0.55	−0.10	−0.11
**ΔQ_800mV H2O2_**	−0.09	−0.19	0.31	0.26	−0.15	−0.35	−0.36	−0.34	−0.13	−0.40	−0.70 *	−0.02	0.26	−0.75 *	0.11	0.10
	**ΔQ_240mV lac_**	**ΔQ_520mV lac_**	**Δ(Q_520mV_-Q_240mV_)_lac_**	**ΔQ_240/800mV lac_**	**ΔQ_800mV lac_**	**ΔQ_240mV 60°C_**	**ΔQ_520mV 60°C_**	**Δ(Q_520mV_–Q_240mV_)_60°C_**	**ΔQ_240/800mV 60°C_**	**ΔQ_800mV 60°C_**	**aOCR H_2_O_2_**	**aOCR lac**	**aOCR 60 °C**	**iOCR H_2_O_2_**	**iOCR lac**	**iOCR 60 °C**
**ΔQ_240mV lac_**	**1.00**	0.99 **	0.39	0.68 *	0.91 **	0.83 **	0.59	0.48	0.63	0.59	0.17	−0.77 *	−0.62	0.29	−0.66	−0.74 *
**ΔQ_520mV lac_**		**1.00**	0.28	0.58	0.92 **	0.84 **	0.59	0.47	0.55	0.60	0.21	−0.70 *	−0.63	0.36	−0.59	−0.72 *
**ΔQ(_520mV_–Q_240mV_)_lac_**			**1.00**	0.68 *	0.02	0.34	0.34	0.32	0.62	0.16	0.28	−0.33	−0.33	0.11	−0.25	−0.31
**ΔQ_240/800mV lac_**				**1.00**	0.48	0.61	0.65	0.63	0.60	0.53	0.10	−0.77 *	−0.48	0.08	−0.76 *	−0.70 *
**ΔQ_800mV lac_**					**1.00**	0.72 *	0.43	0.31	0.50	0.55	−0.06	−0.75 *	−0.54	0.16	−0.67 *	−0.68 *
**ΔQ_240mV 60°C_**						**1.00**	0.88 **	0.79 *	0.68 *	0.89 **	0.39	−0.79 *	−0.83 **	0.39	−0.72 *	−0.90 **
**ΔQ_520mV 60°C_**							**1.00**	0.99 **	0.46	0.92 **	0.48	−0.66	−0.75 *	0.41	−0.66	−0.84 **
**Δ(Q_520mV_–Q_240mV_)_60°C_**								**1.00**	0.36	0.88 **	0.49	−0.58	−0.68 *	0.40	−0.60	−0.78 *
**ΔQ_240/800mV 60°C_**									**1.00**	0.55	0.19	−0.76 *	−0.65	0.14	−0.73 *	−0.64
**ΔQ_800mV 60°C_**										**1.00**	0.26	−0.78 *	−0.79 *	0.21	−0.75 *	−0.89 **
**aOCR H_2_O_2_**											**1.00**	0.07	−0.17	0.89 **	−0.03	−0.05
**aOCR lac**												**1.00**	0.62	0.08	0.95 **	0.85 **
**aOCR 60 °C**													**1.00**	−0.27	0.57	0.90 **
**iOCR H_2_O_2_**														**1.00**	−0.06	−0.10
**iOCR lac**															**1.00**	0.80 **
**iOCR 60 °C**																**1.00**

## Data Availability

Data is contained within the article and Supplementary Material.

## Supplementary Materials

The following are available online at https://www.mdpi.com/article/10.3390/antiox10121943/s1. Table S1: enological analytic characterization of the red wines, Table S2: free SO_2_ levels in the red wines after the different oxidation tests, Figure S1: representation of the scores (wines) (variables—electrochemical parameters for the reference wines (Q) and oxidized wines by the three protocols minus reference wines (ΔQ)) in the plane defined by respectively the first (F1) and second (F2) factor (explained variance: 79.55%).
