# Supplementary material for: Red Wine Oxidation Characterization by Accelerated Ageing Tests and Cyclic Voltammetry
Source: Antioxidants (Basel). 2021 Dec 3;10(12):1943. doi: 10.3390/antiox10121943 (PMC8750522; doi:10.3390/antiox10121943)
Supplement: Supplementary file 1 [file antioxidants-10-01943-s001.zip › antioxidants-1463252-supplementary materials.pdf]

SUPPLEMENTARY INFORMATION

Red wine oxidation characterization by accelerated ageing tests and cyclic voltammetry

Stacy Deshaies, Luca Garcia, Frédéric Veran, Laetitia Mouls, Cédric Saucier and François Garcia

Table S1. Enological analytic characterization of the red wines.

Wine Sample	Alcoholic Strength (%)	Total Acidity (g/L eq. H2SO4)	Volatile Acidity (g/L eq. H2SO4)	pH	Cu (mg/L)	Fe (mg/L)	Total SO2 (mg/L)	Free SO2 After Oxygen Saturation (mg/L) *GC-MS analysis*
R1	14.41 ± 0.01	2.98 ± 0.02	0.55 ± 0.01	3.86 ± 0.01	0.3 ± 0.01	2.55 ± 0.05	22 ± 1.00	14.13 ± 0.22
R2	14.31 ± 0.02	3.00 ± 0.04	0.53 ± 0.01	3.68 ± 0.01	nd	2.1 ± 0.01	≤ 10	2.42 ± 0.03
R3	14.21 ± 0.04	2.78 ± 0.01	0.45 ± 0.01	3.77 ± 0.01	nd	1.2 ± 0.01	nd	0
R4	14.38 ± 0.10	3.00 ± 0.01	0.51 ± 0.01	3.45 ± 0.01	nd	2.85 ± 0.05	nd	0
R5	14.16 ± 0.04	2.67 ± 0.01	0.55 ± 0.01	3.71 ± .01	≤ 0.2	2.95 ± 0.05	≤ 10	1.42 ± 0.03
R6	13.55 ± 0.01	2.86 ± 0.01	0.66 ± 0.01	3.71 ± 0.01	nd	0.75 ± 0.05	29 ± 1.00	5.5 ± 1.2
R7	13.75 ± 0.01	3.05 ± 0.01	0.34 ± 0.01	3.49 ± 0.01	≤ 0.2	2.3 ± 0.01	12 ± 1.00	0
R8	15.42 ± 0.01	3.40 ± 0.01	0.55 ± 0.01	3.42 ± 0.01	0.5 ± 0.01	6.4 ± 0.01	21.5 ± 0.5	0
R9	16.26 ± 0.01	3.13 ± 0.01	0.64 ± 0.01	3.66 ± 0.01	0.3 ± 0.01	1.1 ± 0.01	70.5 ± 0.5	27.1 ± 0.15

Table S2. Free SO level in the red wines after the different oxidation tests.

Wine Sample	Free SO2 (mg/L)
R1 - laccase oxidation	0
R2 - laccase oxidation	0
R3 - laccase oxidation	0
R4 - laccase oxidation	0
R5 - laccase oxidation	0
R6 - laccase oxidation	5.5 ± 1.2
R7- laccase oxidation	0
R8 - laccase oxidation	0
R9 - laccase oxidation	26.7 ± 0.15

Free SO_2 was equal to 0 for both chemical and temperature oxidations.

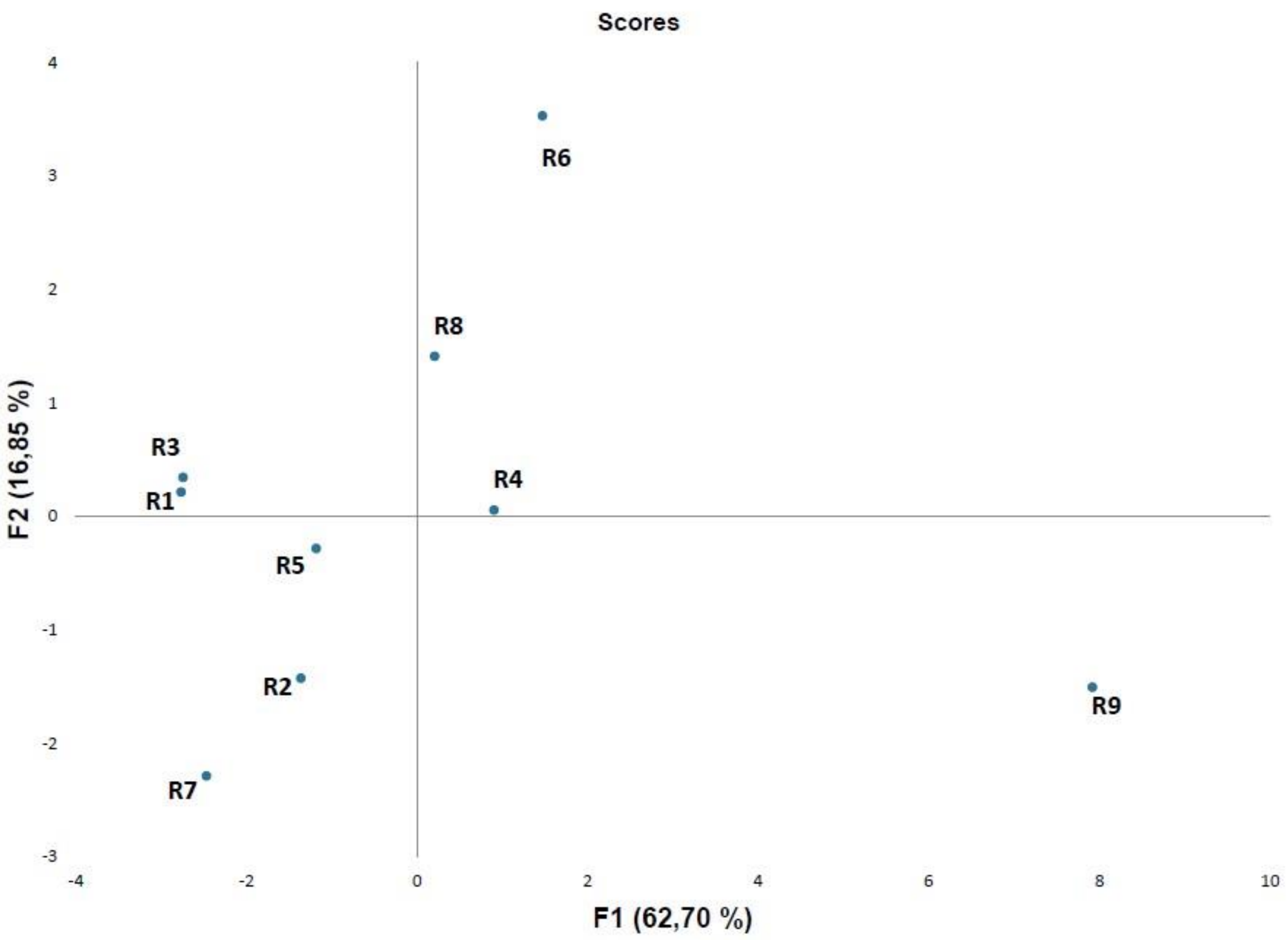

Figure S1. Representation of the scores (wines) (variables – all electrochemical parameters for the reference wines (Q) and for the reference wines minus oxidized wines by the three protocols (ΔQ) in the plane defined by respectively the first (F1) and second (F2) factor (explained variance: 79.55%).
